# Systematic Review and Meta-Analysis of Vesical Imaging-Reporting and Data System (VI-RADS) Inter-Observer Reliability: An Added Value for Muscle Invasive Bladder Cancer Detection

**DOI:** 10.3390/cancers12102994

**Published:** 2020-10-15

**Authors:** Francesco Del Giudice, Martina Pecoraro, Hebert Alberto Vargas, Stefano Cipollari, Ettore De Berardinis, Marco Bicchetti, Benjamin I. Chung, Carlo Catalano, Yoshifumi Narumi, James W. F. Catto, Valeria Panebianco

**Affiliations:** 1 Department of Maternal-Infant and Urological Sciences, “Sapienza” University of Rome, Policlinico Umberto I Hospital, 00161 Rome, Italy; ettore.deberardinis@uniroma1.it; 2 Department of Urology, Stanford University, School of Medicine, Stanford, CA 94305, USA; bichung@uniroma1.it; 3 Department of Radiological Sciences, Oncology and Pathology, “Sapienza”/Policlinico Umberto I, 00161 Rome, Italy; martina.pecoraro@uniroma1.it (M.P.); stefano.cipollari@uniroma1.it (S.C.); marco.bicchetti@uniroma1.it (M.B.); carlo.catalano@uniroma1.it (C.C.); valeria.panebianco@uniroma1.it (V.P.); 4 Department of Radiology, Memorial Sloan Kettering Cancer Center, New York, NY 10065, USA; vargasah@mskcc.org; 5 Departments of Radiology and Health Science, Kyoto Tachibana University, Kyoto 607-8175, Japan; naru21747@gmail.com; 6 Academic Urology Unit, University of Sheffield, Sheffield S10 2TN, UK; j.catto@sheffield.ac.uk

**Keywords:** bladder cancer, VI-RADS, multiparametric magnetic resonance imaging, muscle-invasive bladder cancer, inter-reader agreement, bladder cancer staging

## Abstract

**Simple Summary:**

In our systematic review and meta-analysis of eight observational studies including a total of 1016 patients, we demonstrated excellent pooled inter-observer agreement among Genito-Urinary radiologists when adopting the novel Vesical Imaging-Reporting and Data System (VI-RADS) criteria in the pre-trans-urethral resection of bladder tumor (TURBT) assessment for non-muscle (NMIBC) vs. muscle-invasive bladder cancer (MIBC) detection.

**Abstract:**

The Vesical Imaging-Reporting and Data System (VI-RADS) has been introduced to provide preoperative bladder cancer staging and has proved to be reliable in assessing the presence of muscle invasion in the pre-TURBT (trans-urethral resection of bladder tumor). We aimed to assess through a systematic review and meta-analysis the inter-reader variability of VI-RADS criteria for discriminating non-muscle vs. muscle invasive bladder cancer (NMIBC, MIBC). PubMed, Web of Science, Cochrane, and Embase were searched up until 30 July 2020. The Quality Appraisal of Diagnostic Reliability (QAREL) checklist was utilized to assess the quality of included studies and a pooled measure of inter-rater reliability (Cohen’s Kappa [κ] and/or Intraclass correlation coefficients (ICCs)) was calculated. Further sensitivity analysis, subgroup analysis, and meta-regression were conducted to investigate the contribution of moderators to heterogeneity. In total, eight studies between 2018 and 2020, which evaluated a total of 1016 patients via 21 interpreting genitourinary (GU) radiologists, met inclusion criteria and were critically examined. No study was considered to be significantly flawed with publication bias. The pooled weighted mean κ estimate was 0.83 (95%CI: 0.78–0.88). Heterogeneity was present among the studies (Q = 185.92, d.f. = 7, *p* < 0.001; I2 = 92.7%). Meta-regression analyses showed that the relative % of MIBC diagnosis and cumulative reader’s experience to influence the estimated outcome (Coeff: 0.019, SE: 0.007; *p*= 0.003 and 0.036, SE: 0.009; *p* = 0.001). In the present study, we confirm excellent pooled inter-reader agreement of VI-RADS to discriminate NMIBC from MIBC underlying the importance that standardization and reproducibility of VI-RADS may confer to multiparametric magnetic resonance (mpMRI) for preoperative BCa staging.

## 1. Introduction

The indications for multiparametric magnetic resonance imaging (mpMRI) for bladder cancer (BCa) are recently expanding and have demonstrated reliable accuracy in staging applications [1,2,3,4,5,6]. The Vesical Imaging-Reporting and Data System (VI-RADS) has been introduced to provide preoperative BCa staging and has been prospectively shown to be reliable in assessing the presence of muscle invasion in the pre-TURBT (trans-urethral resection of bladder tumor) setting [7,8]. In particular, the VI-RADS scale consists of a 5-point score for each of the sequences in the acquisition protocol (T2-weighted, Diffusion-weighted imaging [DWI], and Dynamic contrast enhanced [DCE]) combined to obtain an overall sum which predict the likelihood of muscle-invasive disease (muscle invasive bladder cancer (MIBC), i.e., VI-RADS ≥ 3). After its introduction in April 2018, two separate meta-analyses have shown excellent overall pooled diagnostic accuracy for VI-RADS score ≥ 3, with Area Under the Curve (AUC) of 0.94 (95% confidence interval [CI], 0.91–0.95) and 0.93 (95% CI 0.91–0.95), respectively [9,10]. As described in the seminal document [7], the applicability of VI-RADS may extend beyond its intrinsic diagnostic and staging value, leading VI-RADS to act as a predictive tool for several clinical scenarios indicating those non-muscle invasive bladder cancer (NMIBC)’s for secondary resection (Re-TURBT) or as a radiation-free modality to monitor radiological response of MIBC patients eligible for neoadjuvant systemic regimens [8,11,12]. One of the most important and underappreciated aspect of VI-RADS is to provide a systematic and standardized approach to define the risk of muscle invasion. VI-RADS should not therefore be interpreted as a mpMRI competitor for BCa staging, but its complementary development to fill the inter-rater variability gap among radiologists, that in the past prevented the widespread adoption of MRI due to lack of uniform systems and expertise resulting in divergent MRI performance, in different radiology settings. The aim of the present systematic review and meta-analysis is therefore to cumulatively report inter-reader agreement estimate across the most updated available literature on VI-RADS for NMI vs. MIBC discrimination.

## 2. Materials and Methods 

This systematic review and meta-analysis was conducted according to Preferred Reporting Items for Systematic Reviews and Meta-Analyses (PRISMA) guidelines [13]. A research question was established based on the Patient-Index Test-Comparator-Outcome-Study design (PICOS) criteria as the following: what is overall inter-reader agreement among genitourinary radiologists applying VI-RADS for NMIBC from MIBC discrimination? Furthermore, our goal was to compare current evidence within available population-based retrospective and prospective cohort studies. In particular, we determined the pooled Cohen’s κ and/or Intraclass correlation coefficients (ICCs) among radiologists with different level of experiences and genitourinary (GU)-specialized volume institutions. 

### 2.1. Evidence Acquisition 

**Figure 1:**
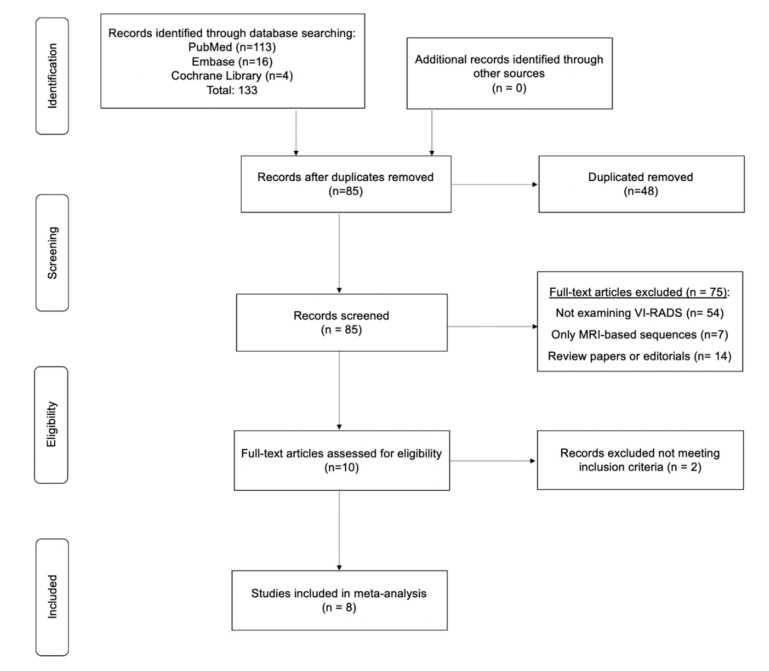
Preferred Reporting Items for Systematic Reviews and Meta-Analyses (PRISMA) flow diagram.

We performed a systematic review of the literature in PubMed, Web of Science, Embase, and Cochrane from inception to 30 July 2020, without language restriction, to identify studies that examined the implementation of pre-TURBT VI-RADS scoring criteria for BCa staging purposes and evaluated the extent of agreement between the radiologists involved. The reference lists of the included studies were also screened for relevant articles. Only original population-based prospective and retrospective cohort studies were included and critically evaluated (Level of Evidence: II and III-a). Case reports, abstracts, and meeting reports were excluded from the analysis. Search terms included but were not limited to: bladder cancer, AND Vesical Imaging-Reporting and Data System or VI-RADS AND multiparametric magnetic resonance imaging or mpMRI AND inter-reader agreement or inter-rater variability AND bladder cancer clinical staging AND radiologists agreement AND preoperative imaging modalities; secondary fields: non-muscle invasive bladder cancer and magnetic resonance imaging; muscle-invasive bladder cancer and magnetic resonance imaging; VI-RADS diagnostic accuracy and inter-reader agreement; bladder cancer stage discrimination; rater characteristics; population-based prospective cohort studies, population-based retrospective cohort studies. A flow diagram and comprehensive list of search terms has been summarized in Figure 1 and Table S1, respectively.

### 2.2. Selection of the Studies and Criteria of Inclusion

Entry into the analysis was restricted to data collected from original articles that examined patients with primary and/or recurrent BCa diagnosis, which assessed final BCa extension through surgical specimen both from TURBT/Re-TURBT or radical cystectomy (RC), and that aimed to report inter-raters performance of VI-RADS for NMI vs. MIBC discrimination. Moreover, only those studies including sufficient data to retrieve inter-reader agreement determination for the outcome of interest (VI-RADS score) and which assessed both “per-patient” and/or “per-lesion” analysis were considered eligible for further consideration. Additionally, studies were considered eligible if all readers were board-certified radiologists with at least 5 years of experience in the Genito-urinary (GU)-MRI imaging, at least two readers were actively involved in the imaging acquisition and revision, and if VI-RADS imaging protocol was followed as previously described according to the original document [7].

Articles were excluded if they met one or more of the following criteria: inadequate information for data extraction or quality assessment; inclusion of study population consisting of <20 patients; presented outcomes which dealt with other topics (e.g., inter-reader agreement used to assess single MRI-sequences without VI-RADS, and VI-RADS score for each MRI sequence was provided but an overall VI-RADS score was not tested for inter-reader variability among radiologists to determine MIBC).

Three authors (FDG, MP, and BIC) independently screened the titles and abstracts of all articles using predefined inclusion criteria. The full-text articles were examined independently by the four (FDG, EDR, CC, and VP) to determine whether or not they met the inclusion criteria. Final inclusion was determined by consensus of all investigators. Selected articles meeting the inclusion criteria were then critically analyzed. 

The following data were extracted from the included studies by using a standardized form: origin of study (institution and period of enrollment), size of study population, period of time prospectively/retrospectively covered, gold standard for MIBC definition, technical parameters of MRI acquisition (T2WI slice thickness, b values used for DWI, and temporal resolution of DCE MRI), details regarding MRI interpretation (number and experience of readers, and whether they were blinded or not), the VI-RADS cutoff value used for determining MIBC on MRI, outcomes related to diagnostic performance of VI-RADS (sensitivity, specificity, positive and negative predictive value (PPV, NPV)), and finally patient’s baseline characteristics and BCa pathological features (e.g., mean age and range of patients, number of tumors, percentage of patients with MIBC, histological subtypes of tumors). 

### 2.3. Assessment of Quality for the Included Studies and Statistical Analysis 

To assess the risk of bias (RoB), all included reports were independently reviewed using the “Quality Appraisal of Diagnostic Reliability (QAREL) Checklist.” Two reviewing authors (FDG and MP) independently assessed sampling bias and the representativeness of subjects and raters as well as rater blinding and the order in which raters or subjects were examined. Additionally, the suitability of the time interval among repeated measurements, whether the test was applied and interpreted appropriately, and finally the statistical analysis of reliability were considered. Publication bias was tested both by visual assessment of the Deeks’ funnel plot and calculation of *p* value using the Deeks’ asymmetry test [14]. The Trim-and-Fill method together with Egger’s regression test were implemented to explore the possible nature of studies “missed” in the single outcome and the relative importance of small-study effect [15]. We compared treatments using pooled weighted Cohen’s κ and 95% Confidence Intervals (CI) considering values as: <0.20 poor reliability, 0.21–0.40 fair, 0.41–0.60 moderate, 0.61–0.80 good, and 0.81–1.00 considered excellent [16]. Sensitivity analyses were performed to assess the contribution of each study to the pooled estimate by excluding individual trials one at a time and recalculating the pooled estimates for the remaining studies. Evaluation for presence of heterogeneity was done using [17]: (1) Cochran’s Q-test with *p* < 0.05 signifying heterogeneity; (2) Higgins I2 test with inconsistency index (I2) = 0–40%, heterogeneity might not be important; 30–60%, moderate heterogeneity; 50–90%, substantial heterogeneity; and 75–100%, considerable heterogeneity. The pooled weighted mean κ estimate was calculated using a random effects model [18]. Our results are graphically displayed as forest plots (all studies or according to sub-groups analysis), with pooled κ indicating overall agreement among readers to discriminate from NMIBC to MIBC using VI-RADS criteria.

Meta-regression analyses were performed using available clinical and radiological variables retrieved among the studies. Pooled weighted κs were plotted against the following available quantitative variables: range of study time screened (months retrospectively or prospectively imputed), months from original VI-RADS description, total number of patients screened, and the overall number of years of experience among the different radiological groups. The point estimates of the weighted mean κ were obtained and plotted with the area of the circles proportional to the inverse of the squared standard errors of the studies included. A second set of meta-regression analyses were conducted implementing categorical variables (sub-groups analysis) such as study design implemented (prospective vs. retrospective), proportion of MIBC detected (<30 vs. >30%), magnetic strength (1.5 vs. 3 T), T2WI slice thickness (3 vs. 4 mm), and VI-RADS cut-off for MIBC (≥4 vs. ≥3). Calculations were accomplished using Stata version 16.1 (Stata Corporation, College Station, TX, USA).

## 3. Results

### 3.1. Search Results

**Figure 2:**
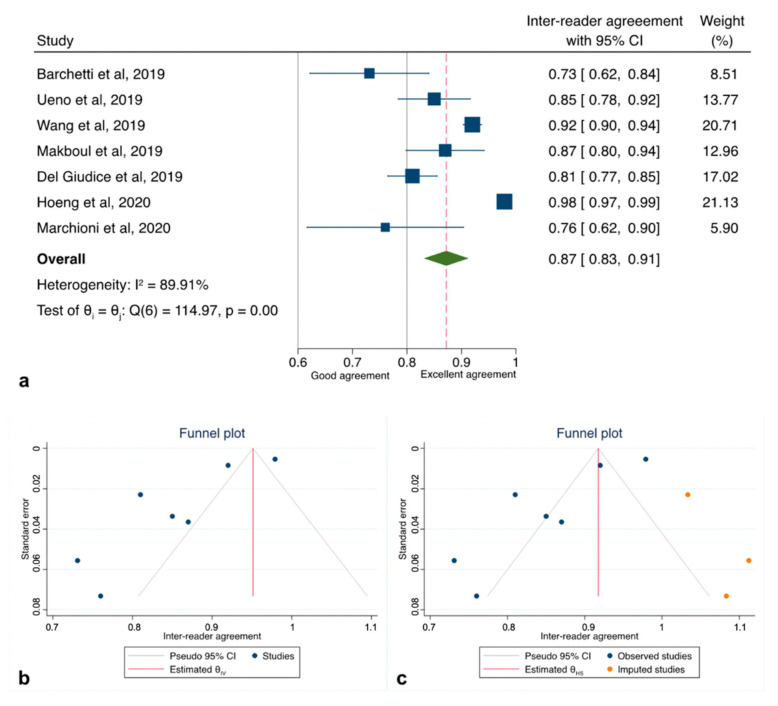
Pooled inter-reader agreement for the studies included in the quantitative analysis. (**a**) Forest plot reporting the pooled inter-reader agreement among the 7 studies analyzed (excluding Sakamoto et al.); (**b**) Deeks’ funnel plot (test for small-study effect, *p* < 0.001); (**c**) “Trim and Fill” method suggesting imputed studies missing to remove asymmetry from the funnel plot.

**Figure 3:**
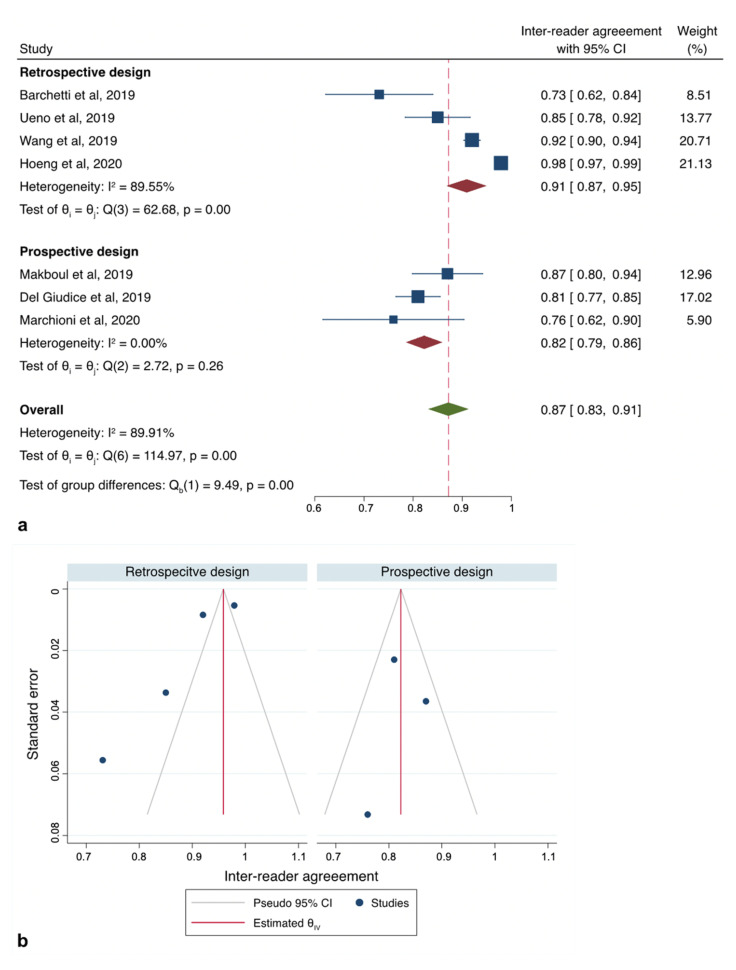
Sub-group analysis according to study design. (**a**) Forest plot reporting the pooled inter-reader agreement according subgroup analysis of the studies (**b**) Deeks’ funnel plot according to “study design” subgroup.

The initial search yielded 133 articles (PubMed: 113; Cochrane Library: 4; and Embase: 16). Forty-eight were excluded as they contained overlapping data or were duplicates appearing in multiple databases. Of the remaining 85, 75 were further excluded because these did not examine VI-RADS (54), contained only MRI-based sequence information (7), were review papers, or editorials (14). Full-text articles were then reevaluated and critically analyzed for the remaining 10 journal references. Within this in-depth review, a further 2 did not meet the inclusion criteria. The remaining 8 studies were included in our review (Figure 1). No study was considered to be seriously flawed as per the “Quality Appraisal of Diagnostic Reliability (QAREL) Checklist.” Studies’ risk to performance bias was overall moderately low with some attrition bias due to incomplete outcome data across all the studies. Individual RoB as well as visual assessment of the Deeks’ funnel plots are illustrated in Table S2, and Figure 2b and Figure 3b.

### 3.2. Location, Design, and Characteristics of the Studies Population

Patient/tumors description, main findings, and study characteristics of the whole studies included are summarized in Table 1. Of the 8 included articles [8,19,20,21,22,23,24,25], 3 were conducted in Italy [8,19,24] and 2 in Japan [20,25], while the remaining were from China [21], Egypt, and Korea [22,23], covering an overall time period between February 2019 and April 2020. Overall, the number of patients included was 1016, varying from 32 to 340 in single experiences, while the prevalence of MIBC ranged from *n* = 6, 10.3% to *n* = 62, 50%. Three study designs [8,22,24] were prospective reporting outcomes from primary or recurrent BCa cases. Out of these, the article from Del Giudice et al. [8] was the sole to analyze a large cohort of patients within prespecified limits of enrolling time (i.e., December 2017–May 2019) and more strict inclusion criteria with regard of previous BCa history and pathology related exclusion features (i.e., no CIS or non-urothelial carcinomas), while the other two experiences [22,24] did not report the same information thus suffering from potential higher RoB. The remaining five studies [19,20,21,23,25] were retrospective, assessing both urothelial and other histology variants of BCa. All studies were performed at single centers using either TURBT or a combination of TUR and partial or RC as the reference standard. No study solely used cystectomy as the reference standard. In all but two of the above studies, confirmatory repeat TUR was performed for appropriate clinical settings (e.g., high-grade NMIBC or insufficient muscle tissue in TUR specimen) to reduce underestimation of MIBC.

### 3.3. Technical Imaging Modalities and Readers Characteristics

All the studies included were considered eligible as per VI-RADS protocol criteria originally described. Table 2 summarizes studies of MRI-specifications and radiological characteristics. MpMRI had been performed prior to TURBT in all included studies except for one, which consisted of a mixture of pre- and post-TUR patients [21]. The majority of the studies implemented at a 3T MRI scanner [8,19,21,23,24,25] with T2-weighted images including slice thickness of 3 mm [8,19,22,24,25] or only 4 mm [20,21,23,25] respectively. Diffusion-weighted images were acquired homogeneously among the studies with high b values of 1000 s/mm^2^ or higher. Although protocols for DCE MRI varied among studies, temporal resolution was sufficient for the depiction of early enhancement of the inner layer followed by tumor enhancement required according to VI-RADS evaluation. Only the study of Sakamoto et al. [25] compared two different MRI protocols within the same study population. Their findings were based on the multiparametric vs. biparametric approach with additional evaluation of apparent diffusion coefficient (ADC) measured on diffusion-weighted magnetic resonance imaging therefore will be subsequently separated from the rest of the cumulative meta-analysis estimates.

Overall, five studies [8,19,21,22,25] included two readers for the VI-RADS scoring process and for definitive consensus in the case of discrepancies assuming the most experienced opinion as the definitive one. In all these cases, a κ estimate with 95%CI or SE was reported. Two studies [23,24] enrolled three different readers with increasing levels of experience. In the experience of Marchioni et al. [24], a third and more experienced reader was consulted only in the case of discrepancies among the first two radiologists, while in the study of Hong et al. [23] all three raters were independently involved in the diagnostic VI-RADS assignment. As a consequence, both in the study of Hong et al. [23] and Ueno et al. [20], who involved five different board-certified GU-radiologists, the measure of inter-variability estimate was the ICCs with 95% CI or SE. In all the examined articles, the readers were blinded to the pathological findings and reported the outcomes implementing a cutoff VI-RADS score of ≥3 to determine MIBC or also ≥4 in five studies [19,20,23,24,25]. The overall cumulative level of radiologist experience was declared in only five studies ranging from a minimum of 24 years to a maximum of 47 years. 

### 3.4. Interobserver Agreement Estimates Among GU Readers

All of the studies reporting data on inter-reader reliability for VI-RADS to detect MIBC reported different associations with a range of mean weighted estimates κ from 0.43 to 0.98. As there was evidence for the presence of substantial heterogeneity between the studies: Q = 185.92 (d.f. = 7), *p* < 0.001; I2 = 92.7%, we reported results according to a random-effects model registering pooled Ƙ estimates of 0.83 (95%CI: 0.78–0.88) (Figure S1a). However, since the study of Sakamoto et al. [25] was flawed by a significantly higher RoB due to substantial differences in the acquisition protocol, we therefore repeated our analysis with only seven studies [8,19,20,21,22,23,24], slightly reducing heterogeneity: Q = 114.97 (d.f. = 6), *p* < 0.001; I2 = 89.9% and obtaining a cumulative κ estimates of 0.87 (95%CI: 0.83–0.91) (Figure 2a). Exploring heterogeneity among the articles, sub-group analysis set for study design (retrospective vs. prospective) revealed the presence of the highest heterogeneity among those with a retrospective design (*p* < 0.001; I2 = 89.5%) rather than those with a prospective (*p* < 0.26; I2 = 0%) (Figure 3 and Table 3).

Inspection of the funnel plot suggested that for all the 7 studies together, there was a significant small-study effect with two studies tending to have a higher outliner estimates as depicted in Figure 2b. Egger’s regression test showed indeed significant small-study effect (*p* < 0.001) whilst the “Trim and Fill” method suggested that three “missing” studies would need to be included to remove asymmetry from the funnel plot (Figure 2c). With these hypothetical studies included, the pooled inter-reader agreement having accounted for publication bias was estimated to be 0.92 (95% CI: 0.88–0.96). Funnel plot inspection grouped for study design graphically displayed the existing asymmetry between retrospective and prospective analyses (Figure 3b).

**Figure 4:**
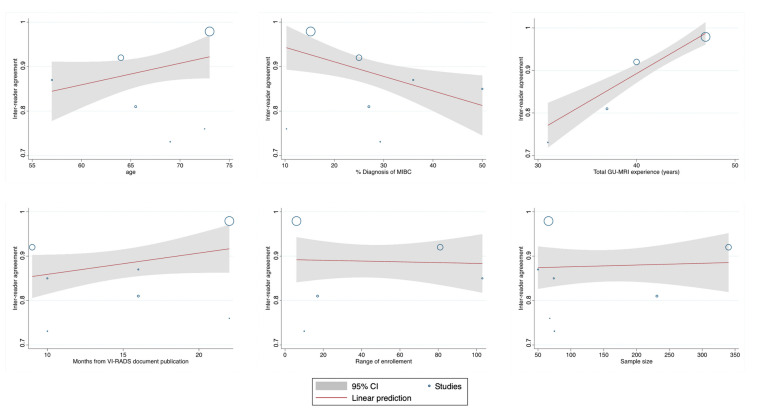
Meta-regression plots of the quantitative variables retrieved among the studies included in the analysis.

A meta-regression summary and plots have been reported in Table 4 and Figure 4. As possibly expected, we found a negative independently significant trend for the quantitative % of MIBC detected in each single study and final agreement (Coeff: −0.019, SE = 0.007; *p* = 0.003), while overall years of MRI experience among the different groups revealed an increased association in the measure of ascertainment (Coeff: 0.036, SE = 0.009; *p* < 0.001).

## 4. Discussion

The adoption of any of the available “Xxx Imaging-Reporting and Data System” for a variety of malignancies, including breast cancer, liver cancer, lung cancer, and head and neck cancers have been adopted worldwide and implemented among radiologists to achieve a reproducible common language to standardize diagnostic interpretation. In urologic oncology, mpMRI of the pelvis has changed the paradigm in the detection, characterization, and management of prostate cancer, including allowing for the ability of targeted biopsies, in refining treatment planning and patient selection for active surveillance, and assessment of post-treatment effects [26,27]. However, the interpretation of mpMRI remains difficult and with substantial inter-reader variability that had led to the development of an original (v.1) and then an updated version (v.2 and 2.1) of the Prostate Imaging Reporting and Data System (PI-RADS) [27,28,29]. All these efforts have led the European Association of Urology (EAU) Guidelines to strongly recommend adherence to PI-RADS guidelines for mpMRI acquisition and interpretation [30]. While we acknowledge the existence of two very separate diagnostic objectives between PI-RADS and VI-RADS, nevertheless, these criteria certainty share the common aim of pursuing a higher reliability among readers for the diagnostic outcomes if compared to a merely subjective interpretation of the MRI sequences. 

Similarly, preoperative diagnostic tools such as computer tomography (CT) and MRI are available for clinical stage determination of BCa, but still lack a high degree of reliability. The accuracy of MRI for primary BCa staging varies indeed from 73% to 96%, according to various studies [1,4,31]. With the aim of standardizing staging, diagnosis, and eventually, therapeutic response to neoadjuvant systemic regimens, the VI-RADS scoring system has been recently introduced and validated through a consensus-driven approach to reproducible imaging and reporting [7]. 

Although it is relatively novel, a growing body of evidence from both retrospective and prospective studies is becoming available, systematically testing VI-RADS for diagnostic applications both for preoperative risk stratification and assessment of therapeutic response post chemotherapy [8,11,12]. Two recent meta-analyses describe the ability of VI-RADS to differentiate between superficial and muscle-invasive disease in a preoperative setting [9,10]. As expected, even if AUC values were similarly high when setting both threshold of ≥3 or a ≥4 for MIBC, the sensitivity and the positive likelihood ratio were found higher for this last one thus delineating two separate plausible profiles to interpret VI-RADS related on one hand to muscle status prediction or on the other, to preoperative assessment before TURBT and eventually RC. 

However, none of the above studies has quantitatively assessed the diagnostic reliability among readers of VI-RADS to predict MIBC. Ours is the first meta-analysis of the currently available evidence. Encouragingly, we found that the pooled inter-agreement estimates ranged from good to excellent in accordance with the different studies enrolled. We readily admit the existence of some heterogeneity among the outcomes reported. However, we would partly attribute this to different study designs among the eligible studies (retrospective vs. prospective). At subgroup analysis, the retrospective studies were indeed found to be influenced by an over-estimating effect upon inter-reader agreement due to the existence of some potential bias. On the other hand, some of the heterogeneity could be also attributed to the intrinsic limitations of the Cohen’s Kappa meta-analysis. Although all the studies relied on the original VI-RADS publication for scoring [7] and were therefore significantly comparable in terms of cohort characteristics, positivity threshold (score ≥3), and reported similar MIBC prevalence, each single Kappa value might not be considered as equivalent to a population parameter, thus the derived variance weighted mean estimated would be possibly influenced by the marginal distributions. Standardizing the interpretation and reporting approach along with continued good predictive performance of the VI-RADS system is the basis of the overall quality of the current meta-analysis and furthermore will represent the additional opportunities for improving upon the currently suboptimal TUR staging algorithm which can understage a significant number of cases, especially when the muscularis propria has not been sampled [32,33]. 

Currently, the reproducibility of VI-RADS versus bladder MRI without VI-RADS has become a debated issue in the radiologist’s and urologist’s community. To date, there are no data showing differences in inter-readers’ agreement comparing MRI using VI-RADS and MRI alone. However, the controversy is wrongly based on the assumption of comparing VI-RADS vs. MRI. VI-RADS is a scoring system developed to standardize MRI interpretation and reporting that has proved to be reliable as score supporting the role of MRI in the diagnosis of MIBC. As such, it is unlikely that VI-RADS and MRI alone show very different performance; however, MRI does not allow to stratify patient prognostic risk according to muscle invasiveness likelihood. In fact, VI-RADS has practical implication in the management of treatment of naïve patients undergoing MRI before TURBT for staging purposes, that MRI alone would not have. Authors have suggested the role of VI-RADS as scoring that might tailor therapeutic planning according to patients’ risk as per precision personalized medicine, lowering under- and over-treatment and overall costs [5,8]. 

MpMRI of the bladder with VI-RADS scores might become as a tool for initial hematuria work-out, prediction of tumor extent, response to systemic therapy, as well as a clinical predictor for perioperative outcomes. This could lead to the developments of diagnostic pathways to early identify prognostic features and novel nomograms among selected patient’s categories. Accordingly, the Bladder Path trial (Available online: https://www.birmingham.ac.uk/research/crctu/trials/bladder-path/index.aspx, October 2020), where TURBT has been investigated for being substituted by mpMRI after bladder biopsy demonstrated the presence of BCa, is moving forward to prove the safety possibility for a more rapid and immediate radical intervention in patients with muscle-invasive disease according to mpMRI findings. Moreover, our group has recently identified VI-RADS score 5 with the highest accuracy to predict locally advanced BCa at RC (≥pT3) as well as identified score 5 as an independent unfavorable predictor for relevant delayed time to cystectomy (i.e., >3 months from TURBT diagnosis of MIBC; odds ratio [OR] 2.81, 95% CI: 1.20–6.62) [34]. All of these future clinical perspectives may lead VI-RADS to become a user-friendly tool for preoperative loco-regional staging as well as a reliable predictor aiding to drive the urological decision-making process for specifically selected BCa categories. 

Although all such promising clinical implications, van der Heijden and Witjes have reasonably and rightly addressed the lack of data regarding reproducibility of the scoring system among different centers warranting the need of multicenter studies [35].

Our results suggest that VI-RADS offer a standard methodology for radiologists in the acquisition, interpretation, and reporting of MRI, nonetheless several limitations warrant mentioning. First, the number of included studies was relatively small (*n* = 8) and there was some heterogeneity among the included studies. Nevertheless, the overall number of patients included was greater than 1000, which mitigates any potential statistical power issues. We also found that greater experience with VI-RADS was the most significant predictor of inter-rater reliability, which could create differential results amongst those tertiary centers most familiar with the system. A higher percent of MIBC prevalence among the studies also corresponded to lower agreement, which is plausible considering the direct clinical and psychological implication to address to a patient when such a diagnosis is presumed. Finally, we admit that possible confounding from other factors, such as time from VI-RADS development or technical aspects (i.e., magnet strength), could occur which will require further exploration in future analyses.

## 5. Conclusions

VI-RADS is a promising diagnostic tool characterized by excellent inter-observer variability in pre-TURBT identification of patients with MIBC. Although this meta-analysis represents the first initial proof of significant agreement of VI-RADS for BCa staging among radiologists working into GU-MRI dedicated imaging institutions and with a significant overall high level of expertise, additional larger, multicenter experiences will be necessary to confirm this initial promising trend. In particular, technical aspects such as the role of magnetic field strength or slice thickness as well as the influence of tertiary compared to non-tertiary medical centers will need to be further explored to confirm whereas mpMRI and VI-RADS can safely be addressed in routine clinical practice to evaluate the presence of muscle invasiveness before a staging/therapeutic TUR procedure.

## Figures and Tables

**Table 1:** Clinical and demographic characteristics of the studies enrolled in the systematic review and meta-analysis. SD, Standard Deviation, IQR, Inter Quartile Range; MIBC, Muscle Invasive Bladder Cancer; TUR, Trans-Urethral Resection; RC, Radical Cystectomy; NA, Not Applicable; PC, Partial Cystectomy.

Author, Year, Location	Period (Range of Time)	Study Design	Sample Size	N. of Lesions Assessed	Age (Year) Mean (SD)/Range (IQR/Range)	T-Stage Assessed Range	MIBC n, % ≥T2	Tumor Histology % Urothelial	Reference Standard	MRI Interval	Level of Evidence
Barchetti et al. [19], 2019, Italy	September 2017–July 2018	Retrospective	75	75	69 (62–78)	Tis–T3a	22, 29.3%	100	TUR	<6 wk	III-a
Ueno et al. [20], 2019, Japan	January 2010–August 2018	Retrospective	74	74	NA	Tis–≥T2	37, 50%	95.9	TUR	NA	III-a
Wang et al. [21], 2019, China	November 2011–August 2018	Retrospective	340	340	64 (27–87)	Ta–T4	85, 25%	100	TUR, PC, RC	<2 wk	III-a
Makboul et al. [22], 2019, Egypt	NA	Prospective	50	50	57.2	T1–T4	18, 36%	NA	TUR	NA	II
Del Giudice et al. [8], 2019, Italy	September 2017–May 2019	Prospective	231	231	65.5 (47–79)	Ta–T4	62, 27%	100	TUR, RC	<6 wk	II
Hong et al. [23], 2020, Korea	July 2018–January 2019	Retrospective	32	66	73 (50–90)	NA	10, 15.2%	NA	TUR, RC	<2 wk	III-a
Marchioni et al. [24], 2020, Italy	NA	Prospective	38	68	72.5 (66.5–81.0)	Ta–T3	7, 10.3%	NA	TUR	NA	II
Sakamoto et al. [25], 2020, Japan	January 2013–September 2018	Retrospective	176	176	73 (30–95)	Ta–≥T2	46, 26%	100	TUR	NA	III-a

**Table 2:** Radiological and MRI-specific characteristics of the studies enrolled in the systematic review and meta-analysis. T2WI, T2-Weighted Imaging; DWI, Diffusion Weighted Imaging; DCE, Dynamic Contrast Enhanced; ICC: interclass correlation coefficient; NA, Not Applicable.

Author, Year, Location	Readers No.	Measure of Inter-Agreement	Radiology Institution	Consensus	Cumulative Experience Years	Blinding to Clinical History	Magnet Strength Tesla	T2WI Slice Thickness mm	DWI b Values s/mm^2^	DCE MRI Temporal Resolution
Barchetti et al. [19], 2019, Italy	2	Cohen’s Kappa	same	Consensus	31	Yes	3	3–4	0–800–1000–2000	Every 5 s
Ueno et al. [20], 2019, Japan	5	ICC	same	Independent	NA	Yes	1.5; 3	4	0–1000	At 40, 80, 120, 160, 200 s
Wang et al. [21], 2019, China	2	Cohen’s Kappa	same	Consensus	40	Yes	3	4	0–1000	5 acquisitions between 20 and 131 s
Makboul et al. [22], 2019, Egypt	2	Cohen’s Kappa	same	Consensus	NA	Yes	1.5	3	0, 400, 800, 1000	At 20, 70, 180 s
Del Giudice et al. [8], 2019, Italy	2	Cohen’s Kappa	same	Consensus	37	Yes	3	3–4	0, 800, 1000, 2000	Every 5 s
Hong et al. [23], 2020, Korea	3	ICC	same	Consensus	47	Yes	3	4	0–50–800–1000	6 acquisition every 30 s
Marchioni et al. [24], 2020, Italy	3	Cohen’s Kappa	same	Independent	NA	Yes	3	3–4	0–600–1000–1500–2000	every 31.2 s for 3.2 min
Sakamoto et al. [25], 2020, Japan	2	Cohen’s Kappa	primary and affiliated	Consensus	24	Yes	3	4–5; 3.5	0–1000; 0–2000	NA

**Table 3:** Meta-regression analysis according to subgroup stratification; CI, Confidence Interval.

Subgroup	Covariate	N. of Studies	Meta Regression (Pooled Agreement)			
			Inter-Reader	95% CI		*p* Value
Sample size	≤100	5	0.89	0.84	0.93	0.74
	>100	2	0.87	0.82	0.93	
MIBC proportion	≤30	5	0.88	0.83	0.93	0.57
	>30	2	0.86	0.81	0.91	
Study design	Retrospective	4	0.91	0.87	0.95	<0.001
	Prospective	3	082	0.79	0.86	
Number of readers	2	4	0.85	0.81	0.90	0.01
	>2	3	0.93	0.89	0.97	
Magnetic strength	1.5 T used	2	0.86	0.81	0.91	0.57
	3.0 T only	5	0.88	0.83	0.93	
VI-RADS cutoff score	≥3	6	0.88	0.84	0.92	0.12
	≥4	1	0.76	0.62	0.90	

**Table 4:** Meta-regression analysis quantitative variables retrieved among the studies included in the analysis. SE; Standard Error; CI, Confidence Interval; GU, Genito-Urinary.

Quantitative Covariates	Simple Meta-Regression					Multivariable Meta-Regression				
	Coef.	SE	*p* Value	95% CI		Coef.	SE	*p* Value	95% CI	
Sample Size. *n*	0.00004	0.00016	0.801	−0.00026	0.00034	-	-	-	-	-
Mean age (years)	0.004846	0.00306	0.113	−0.00115	0.01084	-	-	-	-	-
% ≥T2	−0.00327	0.00132	0.013	−0.00585	−0.00069	−0.019	0.007	0.003	0.007	0.032
Mo. from VI-RADS publication	0.00480	0.00312	0.124	−0.00131	0.01090	-	-	-	-	-
Range of enrollment	−0.00009	0.00047	0.854	−0.00102	0.00084	-	-	-	-	-
Total GU-MRI experience	0.01351	0.00216	0.025	0.00419	0.02282	0.036	0.009	0.001	0.019	0.053

## Supplementary Materials

The following are available online at https://www.mdpi.com/2072-6694/12/10/2994/s1, Figure S1: Pooled inter-reader agreement for the studies included in the initial qualitative analysis. Table S1: Comprehensive list of search terms for primary and secondary fields, Table S2: Risk of bias assessment according to Quality Appraisal of Diagnostic Reliability (QAREL) Checklist.

## References

[1] Huang L., Kong Q., Liu Z., Wang J., Kang Z., Zhu Y. (2018). The Diagnostic Value of MR Imaging in Differentiating T Staging of Bladder Cancer: A Meta-Analysis. Radiology.

[2] Giannarini G., Petralia G., Thoeny H.C. (2012). Potential and limitations of diffusion-weighted magnetic resonance imaging in kidney, prostate, and bladder cancer including pelvic lymph node staging: A critical analysis of the literature. Eur. Urol..

[3] Panebianco V., De Berardinis E., Barchetti G., Simone G., Leonardo C., Grompone M.D., Del Monte M., Carano D., Gallucci M., Catto J. (2017). An evaluation of morphological and functional multi-parametric MRI sequences in classifying non-muscle and muscle invasive bladder cancer. Eur. Radiol..

[4] Caglic I., Panebianco V., Vargas H.A., Bura V., Woo S., Pecoraro M., Cipollari S., Sala E., Barrett T. (2020). MRI of Bladder Cancer: Local and Nodal Staging. J. Magn. Reson. Imaging.

[5] Panebianco V., Del Giudice F., Leonardo C., Sciarra A., Catalano C., Catto J.W. (2020). VI-RADS Scoring Criteria for Alternative Risk-adapted Strategies in the Management of Bladder Cancer During the COVID-19 Pandemic. Eur. Urol..

[6] Pecoraro M., Takeuchi M., Vargas H.A., Muglia V.F., Cipollari S., Catalano C., Panebianco V. (2020). Overview of VI-RADS in Bladder Cancer. Am. J. Roentgenol..

[7] Panebianco V., Narumi Y., Altun E., Bochner B.H., Efstathiou J.A., Hafeez S., Huddart R., Kennish S., Lerner S., Montironi R. (2018). Multiparametric magnetic resonance imaging for bladder cancer: Development of VI-RADS (Vesical Imaging-Reporting and Data System). Eur. Urol..

[8] Del Giudice F., Barchetti G., De Berardinis E., Pecoraro M., Salvo V., Simone G., Sciarra A., Leonardo C., Gallucci M., Catalano C. (2019). Prospective assessment of Vesical Imaging Reporting and Data System (VI-RADS) and its clinical impact on the management of high-risk non-muscle-invasive bladder cancer patients candidate for repeated transurethral resection. Eur. Urol..

[9] Woo S., Panebianco V., Narumi Y., Del Giudice F., Muglia V.F., Takeuchi M., Ghafoor S., Bochner B.H., Goh A.C., Hricak H. (2020). Diagnostic performance of vesical imaging reporting and data system for the prediction of muscle-invasive bladder cancer: A systematic review and meta-analysis. Eur. Urol. Oncol..

[10] Luo C., Huang B., Wu Y., Chen J., Chen L. (2020). Use of Vesical Imaging-Reporting and Data System (VI-RADS) for detecting the muscle invasion of bladder cancer: A diagnostic meta-analysis. Eur. Radiol..

[11] Pecoraro M. (2020). Multiparametric Magnetic Resonance as an accessible tool to evaluate response to neoadjuvant chemotherapy in non-metastatic muscle invasive bladder cancer (MIBC). Eur. Congr. Radiol..

[12] Necchi A., Bandini M., Calareso G., Raggi D., Pederzoli F., Farè E., Colecchia M., Marandino L., Bianchi M., Gallina A. (2020). Multiparametric magnetic resonance imaging as a noninvasive assessment of tumor response to neoadjuvant pembrolizumab in muscle-invasive bladder cancer: Preliminary findings from the PURE-01 study. Eur. Urol..

[13] Liberati A., Altman D.G., Tetzlaff J., Mulrow C., Gøtzsche P.C., Ioannidis J.P., Clarke M., Devereaux P., Kleijnen J., Moher D. (2009). The PRISMA statement for reporting systematic reviews and meta-analyses of studies that evaluate health care interventions: Explanation and elaboration. J. Clin. Epidemiol..

[14] DerSimonian R., Laird N. (1986). Meta-analysis in clinical trials. Control. Clin. Trials.

[15] Duval S., Tweedie R. (2000). Trim and Fill: A simple funnel-plot-based method of testing and adjusting for publication bias in meta-analysis. Biometrics.

[16] Sun S. (2011). Meta-analysis of Cohen’s kappa. Heal. Serv. Outcomes Res. Methodol..

[17] Higgins J.P., Thompson S.G., Deeks J.J., Altman D.G. (2003). Measuring inconsistency in meta-analyses. BMJ.

[18] Van Houwelingen H.C., Arends L.R., Stijnen T. (2002). Advanced methods in meta-analysis: Multivariate approach and meta-regression. Stat. Med..

[19] Barchetti G., Simone G., Ceravolo I., Salvo V., Campa R., Del Giudice F., De Berardinis E., Buccilli D., Catalano C., Gallucci M. (2019). Multiparametric MRI of the bladder: Inter-observer agreement and accuracy with the Vesical Imaging-Reporting and Data System (VI-RADS) at a single reference center. Eur. Radiol..

[20] Ueno Y., Takeuchi M., Tamada T., Sofue K., Takahashi S., Kamishima Y., Hinata N., Harada K., Fujisawa M., Murakami T. (2019). Diagnostic accuracy and interobserver agreement for the vesical imaging-reporting and data system for muscle-invasive bladder cancer: A multireader validation study. Eur. Urol..

[21] Wang H., Luo C., Zhang F., Guan J., Li S., Yao H., Chen J., Luo J., Chen L., Guo Y. (2019). Multiparametric MRI for Bladder Cancer: Validation of VI-RADS for the Detection of Detrusor Muscle Invasion. Radiology.

[22] Makboul M., Farghaly S., Abdelkawi I.F. (2019). Multiparametric MRI in differentiation between muscle invasive and non-muscle invasive urinary bladder cancer with vesical imaging reporting and data system (VI-RADS) application. Br. J. Radiol..

[23] Hong S.B., Lee N.K., Kim S., Son I.W., Ha H.K., Ku J.Y., Kim K.H., Park W.Y. (2020). Vesical Imaging-Reporting and Data System for Multiparametric MRI to Predict the Presence of Muscle Invasion for Bladder Cancer. J. Magn. Reson. Imaging.

[24] Marchioni M., Primiceri G., Pizzi A.D., Basilico R., Berardinelli F., Mincuzzi E., Castellucci R., Sessa B., Di Nicola M., Schips L. (2020). Could Bladder Multiparametric MRI Be Introduced in Routine Clinical Practice? Role of the New VI-RADS Score: Results from a Prospective Study. Clin. Genitourin. Cancer.

[25] Sakamoto K., Ito M., Ikuta S., Nakanishi Y., Kataoka M., Takemura K., Suzuki H., Tobisu K.-I., Kamai T., Koga F. (2020). Detection of Muscle-Invasive Bladder Cancer on Biparametric MRI Using Vesical Imaging-Reporting and Data System and Apparent Diffusion Coefficient Values (VI-RADS/ADC). Bladder Cancer.

[26] Barrett T., Turkbey B., Choyke P. (2015). PI-RADS version 2: What you need to know. Clin. Radiol..

[27] Weinreb J.C., Barentsz J.O., Choyke P.L., Cornud F., Haider M.A., Macura K.J., Margolis D., Schnall M.D., Shtern F., Tempany C.M. (2016). PI-RADS Prostate Imaging—Reporting and Data System: 2015, Version 2. Eur. Urol..

[28] Barentsz J.O., Richenberg J., Clements R., Choyke P., Verma S., Villeirs G., Rouviere O., Logager V., Fütterer J.J. (2012). ESUR prostate MR guidelines 2012. Eur. Radiol..

[29] Turkbey B., Rosenkrantz A.B., Haider M.A., Padhani A.R., Villeirs G., Macura K.J., Tempany C.M., Choyke P.L., Cornud F., Margolis D.J. (2019). Prostate imaging reporting and data system version 2.1: 2019 update of prostate imaging reporting and data system Version 2. Eur. Urol..

[30] Mottet R.C.N., van den Bergh N., Brier E. EAU Guidelines: Prostate Cancer; EAU Guidelines 2020. https://uroweb.org/guideline/prostate-cancer/.

[31] Barentsz J.O., Jager G.J., Witjes J.A., Ruijs J.H.J. (1996). Primary staging of urinary bladder carcinoma: The role of MRI and a comparison with CT. Eur. Radiol..

[32] Divrik R.T., Şahin A.F., Yildirim Ü., Altok M., Zorlu F. (2010). Impact of routine second transurethral resection on the long-term outcome of patients with newly diagnosed pT1 urothelial carcinoma with respect to recurrence, progression rate, and disease-specific survival: A prospective randomised clinical trial. Eur. Urol..

[33] Shindo T., Masumori N., Kitamura H., Tanaka T., Fukuta F., Hasegawa T., Yanase M., Miyake M., Miyao N., Takahashi A. (2013). Clinical significance of definite muscle layer in TUR specimen for evaluating progression rate in T1G3 bladder cancer: Multicenter retrospective study by the Sapporo Medical University Urologic Oncology Consortium (SUOC). World J. Urol..

[34] Del Giudice F., Leonardo C., Simone G., Pecoraro M., De Berardinis E., Cipollari S., Flammia S., Bicchetti M., Busetto G.M., Chung B.I. (2020). Preoperative detection of VI-RADS (Vesical Imaging-Reporting and Data System) score 5 reliably identifies extravesical extension of urothelial carcinoma of the urinary bladder and predicts significant delayed time-to-cystectomy: Time to reconsider the nee. BJU Int..

[35] Van Der Heijden A.G., Witjes J.A. (2020). Vesical Imaging-Reporting and Data System (VI-RADS) for bladder cancer diagnostics: The replacement for surgery?. Eur. Urol. Oncol..

