# Supplementary material for: Systematic Review and Meta-Analysis of Vesical Imaging-Reporting and Data System (VI-RADS) Inter-Observer Reliability: An Added Value for Muscle Invasive Bladder Cancer Detection
Source: Cancers (Basel). 2020 Oct 15;12(10):2994. doi: 10.3390/cancers12102994 (PMC7602537; doi:10.3390/cancers12102994)
Supplement: Supplementary file 1 [file cancers-12-02994-s001.pdf]

cancers

MDPI

Article

Systematic Review and Meta-Analysis of Vesical Imaging-Reporting and Data System (VI-RADS) Inter-Observer Reliability: An Added Value for Muscle Invasive Bladder Cancer Detection

Francesco, Del Giudice [1,2,*]; Martina Pecoraro [3]; Hebert Alberto, Vargas [4]; Stefano Cipollari [3]; Ettore, De Berardinis [1]; Marco Bicchetti [3]; Benjamin I., Chung [2]; Carlo Catalano [3]; Yoshifumi, Narumi [5]; James WF, Catto [6] and Valeria, Panebianco [3]

[1] Department of Maternal-Infant and Urological Sciences, "Sapienza" University of Rome, Policlinico Umberto I Hospital, 00161 Rome, Italy; francesco.delgiudice@uniroma1.it (F.D.G.), ettore.deberardinis@uniroma1.it (E.D.B.)
[2] Department of Urology, Stanford University, School of Medicine, Stanford, CA 94305, USA; bichung@uniroma1.it (B.I.C.)
[3] Department of Radiological Sciences, Oncology and Pathology, "Sapienza"/Policlinico Umberto I, Rome, Italy, 00161 Rome, Italy; martina.pecoraro@uniroma1.it (M.P.); stefano.cipollari@uniroma1.it (S.C.); marco.bicchetti@uniroma1.it (M.B.); carlo.catalano@uniroma1.it (C.C.); valeria.panebianco@uniroma1.it (V.P.)
[4] Department of Radiology, Memorial Sloan Kettering Cancer Center, New York, NY, 10065 USA; vargasah@mskcc.org (H.A.V.)
[5] Departments of Radiology and Health Science, Kyoto Tachibana University, Kyoto 607-8175, Japan; naru21747@gmail.com (Y.N.)
[6] Academic Urology Unit, University of Sheffield, Sheffield S10 2TN, UK; j.catto@sheffield.ac.uk (J.W.F.C.)
* Correspondence: francesco.delgiudice@uniroma1.it; Tel.: +39-0649974201; Fax: +39-0649978509

Received: 5 September 2020; Accepted: 9 October 2020; Published: date

Supplementary

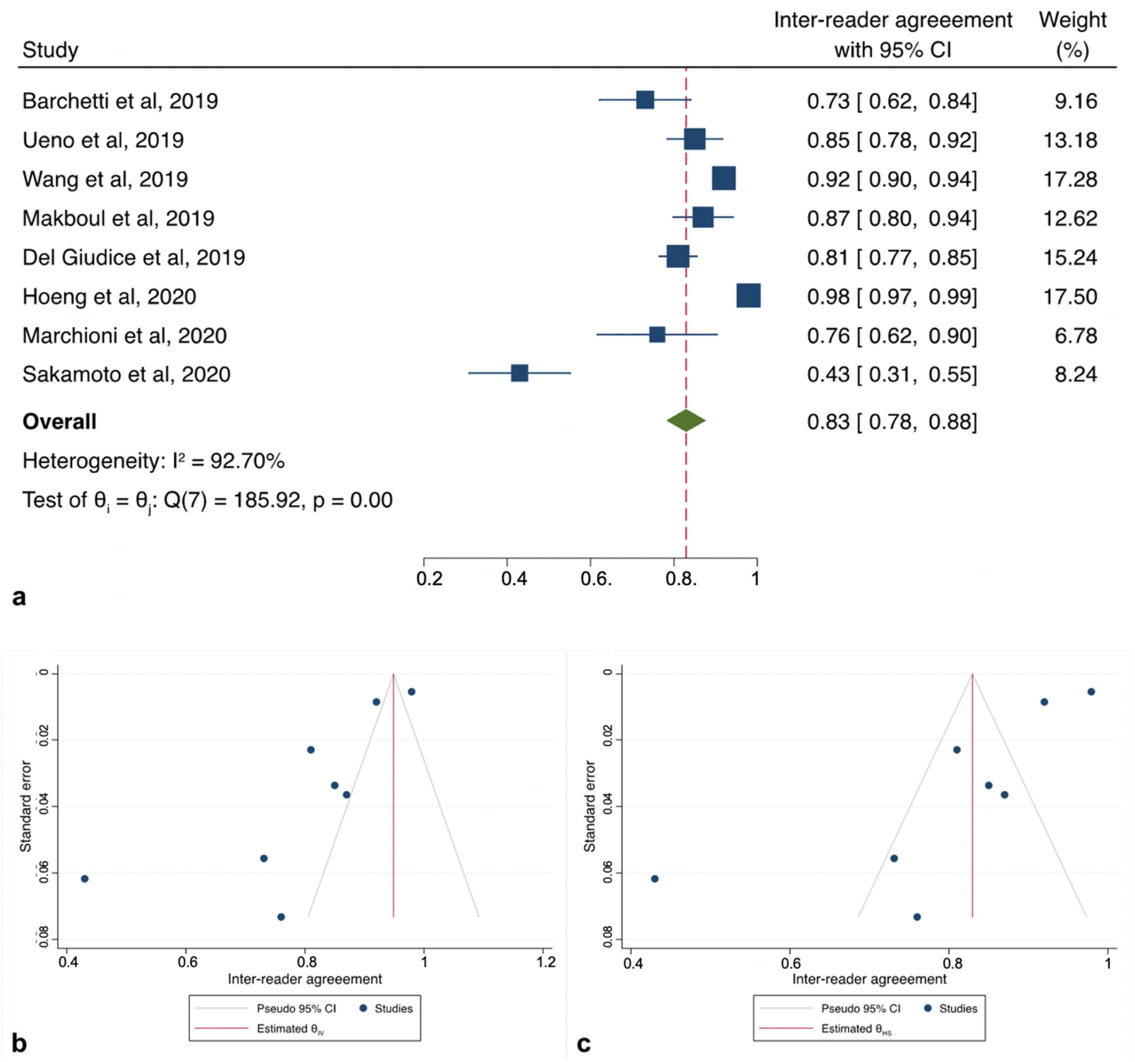

Figure S1. (**a**) Forest plot reporting the pooled inter-reader agreement among the 8 studies (including Sakamoto et al); (**b**) Deeks' funnel plot (test for small-study effect, p<.001); (**c**) 'Trim and Fill' method suggesting imputed studies missing to remove asymmetry from the funnel plot.

Table 1. Comprehensive list of search terms for primary and secondary fields. .

Key words (primary field)	Key words (secondary field)
Bladder Cancer	Non-Muscle Invasive Bladder Cancer and Magnetic Resonance Imaging
Vesical Imaging-Reporting and Data System	Muscle-Invasive Bladder Cancer and Magnetic Resonance Imaging
VI-RADS	VI-RADS diagnostic accuracy and inter-reader agreement
Multiparametric Magnetic Resonance Imaging	Bladder Cancer Stage Discrimination
mpMRI	Rater Characteristics
Inter-reader Agreement	Population-based Prospective Cohort Studies
Inter-rater Variability	Population-based Retrospective Cohort Studies
Inter-reader Reliability	Year of publication
Inter-reader Reproducibility	Radiologist experience
Bladder Cancer Diagnosis	Genitourinary Radiologist
Bladder Cancer Clinical Staging	Diffusion Weighted Imaging
Radiologists Agreement	T2-Weighted Imaging
Preoperative Imaging Modalities	Dynamic-Contrast Enhanced MRI

cancers

Table 2. Risk of bias assessment according to Quality Appraisal of Diagnostic Reliability (QAREL) Checklist.

Author	Year	Item 1	Item 2	Item 3	Item 4	Item 5	Item 6	Item 7	Item 8	Item 9	Item 10	Item 11
Barchetti et al [19]	2019	+	+	+	?	+	+	-	NA	NA	+	+
Ueno et al [20]	2019	+	+	+	+	+	+	+	NA	NA	+	+
Wang et al [21]	2019	?	+	+	+	+	+	+	NA	NA	+	+
Makboul et al [22]	2019	+	+	+	+	+	+	?	NA	+	+	+
Del Giudice et al [8]	2019	+	+	+	+	+	+	?	NA	+	+	+
Hong et al [23]	2020	+	+	+	+	+	+	-	NA	NA	+	+
Marchioni et al [24]	2020	+	+	-	?	+	+	-	NA	NA	+	+
Sakamoto et al [25]	2020	-	+	+	?	+	+	-	NA	NA	-	+

1. Was the test evaluated in a sample of subjects who were representative of those to whom the authors intended the results to be applied?
2. Was the test performed by raters who were representative of those to whom the authors intended the results to be applied?
3. Were raters blinded to the findings of other raters during the study?
4. Were raters blinded to their own prior findings of the test under evaluation?
5. Were raters blinded to the results of the reference standard for the target disorder (or variable) being evaluated?
6. Were raters blinded to clinical information that was not intended to be provided as part of the testing procedure or study design?
7. Were raters blinded to additional cues that were not part of the test?
8. Was the order of examination varied?
9. Was the time interval between repeated measurements compatible with the stability (or theoretical stability) of the variable being measured?
10. Was the test applied correctly and interpreted appropriately?
11. Were appropriate statistical measures of agreement used?

NA: not applicable.
